# Ebola Virus Disease 2013-2014 Outbreak in West Africa: An Analysis of the Epidemic Spread and Response

**DOI:** 10.1155/2015/769121

**Published:** 2015-03-17

**Authors:** Orlando Cenciarelli, Stefano Pietropaoli, Andrea Malizia, Mariachiara Carestia, Fabrizio D'Amico, Alessandro Sassolini, Daniele Di Giovanni, Silvia Rea, Valentina Gabbarini, Annalaura Tamburrini, Leonardo Palombi, Carlo Bellecci, Pasquale Gaudio

**Affiliations:** ^1^Department of Industrial Engineering, University of Rome Tor Vergata, Via del Politecnico 1, 00173 Rome, Italy; ^2^International Master Courses in Protection against CBRNe Events, Department of Industrial Engineering and School of Medicine and Surgery, University of Rome Tor Vergata, Via del Politecnico 1, 00173 Rome, Italy; ^3^Department of Science, University of Rome 3, Viale Marconi 446, 00154 Rome, Italy; ^4^Department of Biomedicine & Prevention, School of Medicine and Surgery, University of Rome Tor Vergata, Via Montpellier 1, 00173 Rome, Italy

## Abstract

The Ebola virus epidemic burst in West Africa in late 2013, started in Guinea, reached in a few months an alarming diffusion, actually involving several countries (Liberia, Sierra Leone, Nigeria, Senegal, and Mali). Guinea and Liberia, the first nations affected by the outbreak, have put in place measures to contain the spread, supported by international organizations; then they were followed by the other nations affected. In the present EVD outbreak, the geographical spread of the virus has followed a new route: the achievement of large urban areas at an early stage of the epidemic has led to an unprecedented diffusion, featuring the largest outbreak of EVD of all time. This has caused significant concerns all over the world: the potential reaching of far countries from endemic areas, mainly through fast transports, induced several countries to issue information documents and health supervision for individuals going to or coming from the areas at risk. In this paper the geographical spread of the epidemic was analyzed, assessing the sequential appearance of cases by geographic area, considering the increase in cases and mortality according to affected nations. The measures implemented by each government and international organizations to contain the outbreak, and their effectiveness, were also evaluated.

## 1. Introduction

Haemorrhagic fever caused by Ebola viruses (EVD, Ebola Virus Disease) is one of the most serious viral diseases currently known, characterized by a high case-fatality rate around 40–50% (that, in some outbreak, reached 90%). Currently, no prophylactic or therapeutic strategies are available [1]: this makes this threat a serious concern to the community. If to date the high case-fatality rate restrict the viral expansion, because most of the outbreaks began and ended in rural areas, with the reaching of the urban areas of West Africa during the epidemic in 2014, the risk of uncontrolled spread became a real concern [2]. For these reasons, the World Health Organization (WHO) in August 8, 2014, declared the EVD outbreak in West Africa a public health emergency of international concern [3].

**Figure 1:**
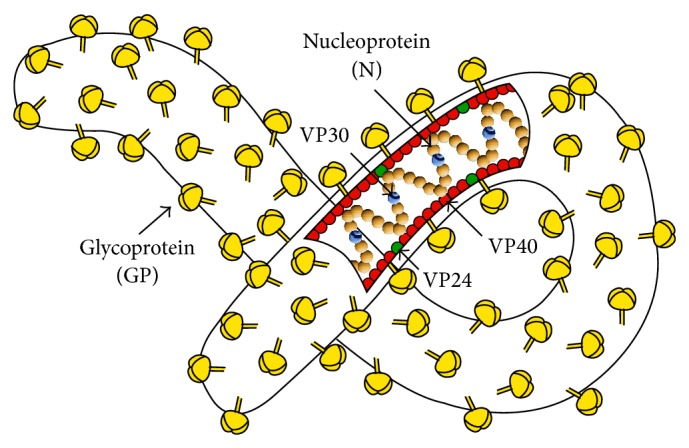
Ebola virus representation. The virion and main proteins are represented. The see-through window in virion shows the negative-sense RNA single-stranded genome covered by nucleoproteins and VP30.

The EVD etiological agent is an enveloped, nonsegmented, RNA negative stranded virus of the family of* Filoviridae* (Figure 1) [4]. The viral particle has a characteristic pleomorphic filamentous morphology (generally ≈1 *μ*m in length; however viral particles lengths up to ≈14 *μ*m were reported in literature [5]) with a constant diameter of 80 nm. The viral genome, approximately 19 kilobases (kb) long, contains seven genes transcribed by the complex of the RNA-dependent RNA polymerase (L and VP35 proteins). The proteins encoded by the viral genome are the N protein, which encapsulates the genome together with the VP30 protein and with the polymerase complex (L and VP35); the VP40 and VP24, which are the matrix proteins; and the GP glycoprotein, which is the VAP (Virus Attachment Protein). GP forms trimeric spikes on the viral envelope mediating the viral adsorption to the cell membrane [4]. Moreover, during the infection Ebola virus produces four soluble glycoproteins: sGP, delta peptide (Δ-peptide), GP_1_, and GP_1,2Δ_. These proteins seem to be involved in viral pathogenesis, particularly targeting cell activation [6].

Ebola virus was first discovered in 1976 when two outbreaks of hemorrhagic fever occurred in northern Zaire (now the Democratic Republic of Congo) and southern Sudan. The outbreaks involved what later proved to be two different Ebola viruses; both were named after the nations in which they were discovered. Since 1976, Ebola virus has appeared sporadically in Africa, with small to large EVD outbreaks. Actually, five Ebola viruses are known: Ebola virus (EBOV, emerged first in Zaire) and Sudan virus (SUDV), discovered in 1976; Reston virus (RESTV), discovered in 1989; Taï Forest virus (TAFV), discovered in 1994; and Bundibugyo virus (BDBV), discovered in 2007 [7]. Only four Ebola virus species, EBOV, SUDV, TAFV, and BDBV, are pathogenic in humans.

Several evidences indicate the Filoviruses as zoonotic, transmitted to humans from ongoing life cycles in animals [8]. Analyses on different animal species during an EVD outbreak seemed to designate particular species of fruit bats as probable virus reservoir [9]. Ebola virus human infection is generally transmitted through close contact with the blood, secretions, organs or other body fluids of infected animals. Person-to-person transmission involves close personal contact (broken skin or mucous membranes) between an infected individual or their body fluids (feces, urine, saliva, or semen) and healthy person. When close contact between uninfected and infected people is avoided or minimized, the number of new Ebola virus infections in humans usually declines. Although in the laboratory the viruses display some capability of infection through small-particle aerosols, airborne spread among humans has not been demonstrated [10]. Burial ceremonies in which mourners have direct contact with the body of the deceased person can also play a role in the spread of Ebola virus [11].

The incubation period is between 2 and 21 days; patients are infectious only in the symptomatic phase, while no infectivity during incubation period is shown. After incubation, the victims develop a febrile illness characterized by headache, muscle and joint pain, and weakness, followed by involvement of the gastrointestinal tract. During the symptomatological phase, hemorrhagic manifestations, in particular on the gastrointestinal tract, represent the major form of clinical manifestation, in addition to a hepatic involvement [12]. More than half of the affected individuals also develop a maculopapular rash associated with erythema. Similarities with symptoms associated with other diseases that are endemic in the same regions cause mistakes in diagnosis, which is easily missed early in the disease course.

Disease diagnosis can be executed only in specific laboratories. In fact EVD can only be confirmed through several types of laboratory tests: (a) antibody-capture enzyme-linked immunosorbent assay (ELISA); (b) antigen detection tests; (c) serum neutralization test; (d) reverse transcriptase polymerase chain reaction (RT-PCR) assay; (e) electron microscopy; (f) virus isolation by cell culture. Samples from patients are an extreme biohazard risk; testing should be conducted under maximum biological containment conditions [11]. In vitro studies have demonstrated that Ebola virus presents a broad cell tropism: monocytes, macrophages, dendritic cells, fibroblasts, endothelial cells, adrenal cortical cells, hepatocytes, and several types of epithelial cells all lend support to replication of Ebola viruses [13].

## 2. EVD Outbreak in West Africa, 2013-2014

The EVD outbreak that hit West Africa since late 2013, caused by EBOV species of Ebola virus, represents an unprecedented event in terms of geographical spread and number of affected individuals. At the date of December 31, 2014, a total of 20200 confirmed cases of EVD were reported. In Guinea, a total of 2707 confirmed cases were reported, including 1708 deaths (case-fatality rate 63,1%); in Liberia a total of 8018 confirmed cases were reported, including 3423 deaths (case-fatality 42,7%); in Sierra Leone a total of 9446 confirmed cases were reported, including 2758 deaths (case-fatality rate 29,2%). In the countries less affected, Nigeria, Mali, and Senegal, respectively, 20, 8, and 1 confirmed cases were reported, including, 8, 6, and 0 deaths. Case-fatality rates for these countries were 40%, 75%, and 0%, respectively [14].

In this work, the geographical spread of the epidemic, from the bursting until the middle of August 2014, and the number of confirmed, suspected, or probable cases and related deaths are presented.

As sources to determine the number of cases and deaths, reports issued by the WHO have been used; progressive reports of duration equal to 10 ± 4 days, dividing the time considered in 15 periods, were considered (Table 1).

**Figure 2:**
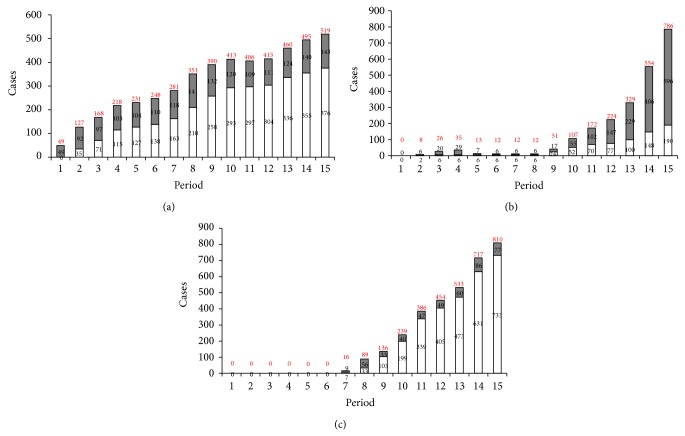
Time trend of compatible EVD cases (confirmed, white bars; probable and suspected, grey bars) in affected countries Guinea (a), Liberia (b), and Sierra Leone (c) according to the periods reported in Table 1. Red numbers over each bar represent the total amount of all compatible cases (confirmed, probable, and suspected) for each period.

**Figure 3:**
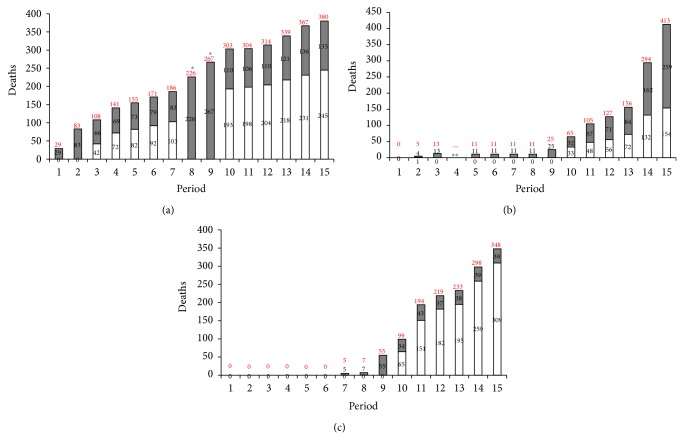
Time trend of compatible EVD deaths (confirmed, white bars; probable and suspected, grey bars) in affected countries Guinea (a), Liberia (b), and Sierra Leone (c) according to the periods reported in Table 1. Red numbers over each bar represent the total amount of all compatible deaths (confirmed, probable, and suspected) for each period. ^*^ in (a) indicates that in the relative WHO reports the distinction between confirmed and unconfirmed cases was not made. ^**^ in (b) indicates that in the relative WHO report notices about the cases progression in Liberia were not reported.

In Figures 2 and 3, the increments for each nation of the compatible (confirmed + probable and suspected) cases and deaths, respectively, are shown. WHO classified as confirmed any suspected or probable case with a positive laboratory result; as probable any suspected case evaluated by a clinician, or any deceased suspected case having an epidemiological link with a confirmed case where it has not been possible to collect specimens for laboratory confirmation; and as suspected any individuals suffering or having suffered from sudden onset of high fever and having had contact with a suspected, probable, or confirmed case, or a dead or sick animal, or any person with sudden onset of high fever and at least three of the EVD symptoms [15].

As it can be seen, while the spread of the outbreak in Guinea, the first affected country, has followed a linear pattern of diffusion and increase in both the emergence of compatible cases (Figure 2(a)) and in related deaths (Figure 3(a)), in countries where the epidemic has had a longer late-onset (Liberia, before, and Sierra Leone, then), the epidemic shows a sharpest increase, both in compatible cases (Figures 2(b) and 2(c)) and then in related deaths (Figures 3(b) and 3(c)). The trends shown in Figures 2 and 3 are currently confirmed over the last reports issued by the WHO, with a dramatic increase of cases in Liberia and Sierra Leone.

Both the structures for the management and treatment of EVD, and the laboratories for clinical and molecular analysis, appear to be more effective in Guinea compared to other affected countries. A substantial lack of capability to respond in Liberia and Sierra Leone, which can justify the large increase in the number of cases from their appearance, which, although delayed in time, in a short period has exceeded the number of cases of Guinea and substantially equaled the number of fatalities, has been highlighted.

At the level of geographical spread, differences in the way of spread were also evidenced; for these reasons, the geographical spread was analyzed.

## 3. Preventive and Control Measures

Although through ecological niche modeling the potential spread of EVD in West Africa was forecasted [16], the outbreak of EVD in Guinea caught all local and international health community unprepared. The first issue was the disease recognition; indeed various endemic diseases, such as malaria and Lassa fever, present many similarities in symptomatology with EVD [17].

Ebola virus diffusion, in the first weeks of outbreak in Guinea, went unnoticed till the appearance of the first serious symptomatology and deaths. Once was identified the threat, Ministry of Health, collaborating with WHO and other partners, made efforts to implement measures to control the outbreak and, most of all, prevent a further spread of the infection [18].

**Figure 4:**
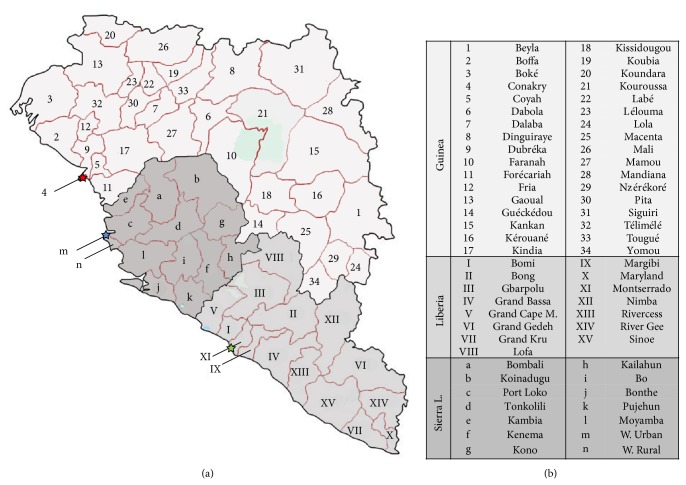
(a) Affected nations (Guinea, Liberia, and Sierra Leone) divided in the respective administrative areas. Guinea, light grey, 34 prefectures, numbers from 1 to 34; Liberia, 15 countries, mild grey, Roman numerals from I to XV; Sierra Leone, dark grey, 14 districts, letters from a to n. Stars indicate the capital cities; red star, Conakry; green star, Monrovia; blue star, Freetown. (b) In the panel the regions in which each country is suborganized are reported.

In order to evaluate the geographical spread of the EVD epidemic, reports issued at the same dates already taken in account to determine the number of cases and deaths by WHO and by the Centers for Disease Control and Prevention (CDC) were used. The affected nations from the bursting until the middle of August 2014 (Guinea, Liberia, and Sierra Leone) were divided in the respective administrative areas, as shown in Figure 4. Guinea includes 33 prefectures, in addition to the capital Conakry. Liberia is divided into 15 countries, while Sierra Leone is divided into 12 districts plus the urban and the rural west areas that divided the capital Freetown into two additional districts.

The geographic spread and the EVD cases in Nigeria were not reported in figures and tables as they were not yet consolidated during the drafting of this paper.

**Figure 5:**
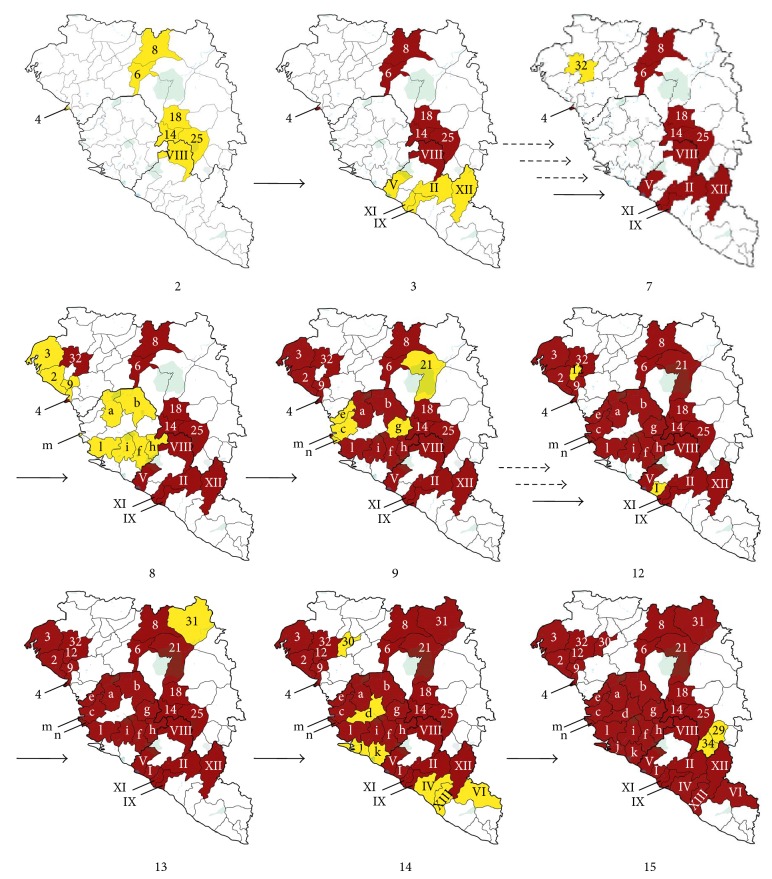
Progressive geographical spread of the EVD outbreak from period 2 to period 15 (for period 1, until the publication of the first WHO report, consolidated geographical details have not been identified). During periods 4, 5, and 6 and during the periods 10-11 there were no progressions in the geographical spread. The periods are reported according to Table 1. Areas barely affected by the epidemic are shown in yellow; the areas already interested with the progression of time are shown in red. Only areas progressively affected were numbered according to administrative areas reported in Figure 4. Solid arrow indicates a spreading into new areas during subsequent periods, according to Table 1. Intermittent arrow indicates no EVD diffusion in new areas for a number of periods according to the arrows amount.

In Figure 5 progressive maps of Guinea, Liberia, and Sierra Leone (from 2 to 15) have shown territories affected by EVD compatible cases (confirmed, probable, or suspected). The data, obtained from reports issued by WHO and CDC, are related to the same periods evaluated to define the geographical spread. By the observation of the maps, it can be highlighted how the spread has followed a different pattern depending on time. Sierra Leone, the last country chronologically extensively affected by EVD, has seen the most rapid spread of the outbreak, with an extension to the whole territory (14 districts) in a reduced period of time (periods 7, 8, and 9; Figure 5). The spread in Guinea seems to have followed a more gradual trend, with a widely distribution during the time. In particular, the affected areas were first localized in the central area and subsequently in the western one, with a progressive involvement of adjacent prefectures to those previously affected (periods 2, 7, 8, 9, 12, 13, 14, and 15). At the date of August 15, 16 prefectures from a total of 34, including the capital Conakry, were affected. The spread in Liberia had a trend concentrated in early periods (periods 2 and 3, Figure 5) and late periods (periods 12 and 14, Figure 5). During the evaluated period, 10 countries from a total of 15 were affected, including a large increase of the cases in the capital Monrovia.

Local and international response have had to face different problems to control the outbreak: (a) health-care operators untrained to manage suspected and confirmed cases; (b) response teams difficulty to trace contacts of probable affected individuals; (c) resources organization following new outbreaks of infection; (d) local community distrust of control and prevention teams [19].

When the outbreak began to spread, the establishment of isolation facilities in the affected areas was one of the first and fundamental steps to control virus diffusion and to provide medical assistance to suspected and confirmed cases. WHO and the Global Outbreak Alert and Response Network (GOARN) deployed experts to support every step of the operational response: surveillance and epidemiology, infection prevention and control, case management, public information, and social mobilization [18]. Fast and precise diagnostic capability to identify infected patients was provided by different laboratories and organization, first of all the* Institut Pasteur* in Dakar, which deployed a mobile laboratory team in Guinea [18].

With the evolution of the outbreak situation, its spread to Guinea's Capital Conakry, and the report of suspected cases in Liberia and Sierra Leone, the need of a strong control and prevention plan became mandatory. WHO alerted all bordering countries to increase surveillance for symptoms consistent with viral hemorrhagic fever and started to train health and community workers to detect, notify, and manage suspected cases [20].

Health-care workers unpreparedness and deficits in the health-care system took to the spreading of infection in hospital between health workers, so training of personnel and efforts to increase awareness on EVD were and are essential steps in the outbreak response plan together with contact tracing. Ministries of Health of affected countries released public communications to increase people awareness on virus transmission and its prevention and to promote collaboration with health teams deployed in the area [21]. Medical supplies and personal protective equipment were and are continuously provided to hospital workers and teams thanks to international partners like WHO, United Nations High Commissioner for Refugees (UNHCR),* Médecins Sans Frontières* (MSF), CDC, and many others [22]. Cross borders meeting between authorities of countries involved in the outbreak have been coordinated with the help of WHO with the aim to find an agreement on a common action plan to reduce the spread of the infection, sharing information on the cross border movement of suspect cases and reinforcing community awareness of EVD. Diagnostic capability was increased thanks to the installation of Real Time Ebola virus-specific PCR and Lassa virus, yellow fever, and Marburg virus PCR in Metabiota Laboratory in Kenema, Sierra Leone [22].

In the attempt of compensating and controlling the spread of the infection and the increase of suspected cases notifications, WHO and international partners deployed constantly new experts to assist local authorities in the various aspects of outbreak response: medical anthropology, clinical case management, data management, surveillance and epidemiology, infection prevention and control, laboratory services, logistics, social mobilization, and resource mobilization [23]. Efforts made by affected countries and international partners seemed to be repaid, during the month of May, showing a decrease in the number of new suspected cases. WHO facilitated participation of national authorities and community leaders to address community resistance and hiding of cases. At the end of May new outbreak point was notified with a high increase of suspected cases in two new districts of Guinea, promptly provided with isolation facilities, and in Sierra Leone from a district sharing borders with Gueckedou district of Guinea [24]. WHO held multilevel teleconference to set up a response plan about new cases notification in Sierra Leone and to arrange resources on the field [24]. As efforts to control and prevent infection propagation kept on, WHO carried on to provide guidance on vigilance insurgence [25, 26].

WHO, with the collaboration of authorities, arranged a high-level meeting in Accra (Ghana) for the Ministers of Health in the subregion to ensure political commitment and cross border collaboration and to settle on a common plan of action to stop the spreading of EVD. At the same time WHO, GOARN, and partners were closely supporting Ministries of Health in deploying additional experts in the various specialties [27]. In the previous reported meeting, with the participation of Ministers of Health and senior health officials from 11 African countries, international partners, EVD survivors, representatives of airlines and mining companies, and donor communities, all agreed on a common strategy action plan to interrupt virus diffusion and to provide a better organization of the resources. Trying to fill all possible gaps discussed, WHO established a subregional centre in Guinea (Conakry) to coordinate and consolidate technical support and resource mobilization to West African countries [28, 29]. With the support of WHO, Guinea, Liberia, and Sierra Leone proceeded in reviewing and updating of respective EVD national response plans to align to the common strategy defined in Accra meeting [30]. WHO started to train community volunteers and supervisors in Liberia and Sierra Leone to conduct contact tracing and provide immediate evacuation and isolation of suspected cases of EVD; in fact contact tracing appears actually to be the best way to stop the spreading of EVD infection [30]. A political delegation of Guinea visited one of the most affected districts (Guéckédou) to build a relationship of trust within the community; most of village leaders pledged their commitment to embrace EVD outbreak response [31]. Authorities agreed that protection of response teams will be assured by law enforcement [31].

In August, during a joint declaration, the Heads of State and Government of the Mano River Union (*Côte d'Ivoire*, Guinea, Liberia, and Sierra Leone) guaranteed to commit additional resources to the outbreak and agreed on common measures, like intercountry level collaboration to focus on cross border regions, enhancing health-care centers treatment, testing, and contact tracing activities in these zones; increasing burials control according to national health regulations; establishment of motivations, treatment, and protection for health personnel; international support to surveillance, contact tracing, case management, and laboratory capacity; intensifying efforts to increase community awareness about EVD; resource research by international partners; improving infection prevention and control protocols [32].

First response in West Africa was focused on treatment of Guéckédou (Guinea), Kenema (Sierra Leone), and Foya (Liberia) as a unified sector, improving of affected country measures, and blocking international spread to other countries [33]. On August 6-7 the Emergency Committee declared Ebola Outbreak in West Africa a “Public Health Emergency of International Concern” [34]. On August 11, WHO organized a panel of medical ethicists, scientific experts, and unqualified people from the affected countries to consider the potential use of unregistered therapies. The panel reached consensus that even if these experimental treatments could have adverse effects and unknown efficacy it is ethical to use them as potential treatment or prevention because of the gravity of the situations and the lack of approved drugs [35]. The experimental drug (ED) ZMapp, developed by Mapp Biopharmaceutical Inc., has been administered to three medical doctors infected with Ebola virus who showed signs of improvement [36]. However, there are no scientific evidences that the health improvement is related to the drug administration (other treated patients died); moreover the quantities of this drug are very limited [37]. WHO does not recommend the application of any travel or trade restrictions except in cases of confirmed or suspected EVD infection or documented contact with cases of EVD [38]. WHO declared in a press conference that air travel from EVD-affected countries is low-risk for Ebola virus spread because virus infection can be transmitted only through contact with bodily fluids [39].

Cases and death counts are exponentially increasing [40]; while bordering countries close their borders, Liberia authorities impose nighttime curfew in the affected district and quarantine zone in West Point, Monrovia [41]. Soldiers and policemen are being employed to face disorders and riots. International partners are trying to provide food supplies to population in quarantine.

The possibility of an effective therapy for EVD is still uncertain: three medics were treated with the ED ZMapp; two of them healed completely and one of them died despite the drug administration [42]; in the meantime Japan scientists proposed to WHO the use of an antiviral effective for influenza infection that could be efficacious for EVD too [43]. Since the worsening of the situation WHO and MSF asked for help and support to any nations which can provide medical equipment supplies, expert staff, and economical resources. WHO encourages all countries to set up active surveillance and emergency response plans [44].

Recently, a suspected case of EVD in Senegal was reported: a person that was identified as a close contact of an EVD infected patient escaped surveillance system and travelled to Senegal to visit his relatives. The patient was previously treated with antimalaria drugs; however the failure of such treatment enforced suspicions of EVD. Sample tests were carried out at the Dakar laboratory [45].

Recently the emerging of a new hemorrhagic fever outbreak was reported in the Democratic Republic of Congo (DRC) which declared an EVD emergency in the region of Djera after that two samples tested positive to Ebola virus infection. Samples analyses evidenced that this outbreak is independent from the one in West Africa: the outbreak reported in the DRC is indeed caused by a variant of EBOV different from the variant that caused the outbreak in West Africa [46].

## 4. Discussion

“Just a few weeks ago, there were only two villages left in Guinea that MSF still had to monitor for ‘contact' people—anyone who had been in contact with confirmed or suspected cases of Ebola. As a result, we were quite hopeful that we were witnessing the end of the epidemic. But then, all at once, we received calls from three different sites in Guinea. Within five minutes, everything had changed.” So spoke MSF's Dr. Hilde de Clerck on July 9, 2014, during an interview about EVD outbreak in West Africa when national and international authorities began to understand they lost control of this epidemic: what seemed to be a limited outbreak with foci only in few districts of Guinea suddenly, in less than a month, became the “largest, most severe, most complex outbreak of Ebola virus disease in history” [35]. The reason beyond the large spread of this disease, which in this moment involves several countries in West Africa and counts a large amount of deaths, is due to a multiplicity of problems that authorities were forced to face to reduce EVD spreading. The current situation is unprecedented; previous EVD outbreaks were contained thanks to early detection and isolation, contact tracing and monitoring, and observance to rigorous protocols of infection prevention and control [35]. Furthermore previous outbreaks were in rural zones or isolated villages that offered quite easy containment (the only urban area previously affected by EVD was Kikwit (DRC), in 1995; however in that case the outbreak remained confined) [47]; in current outbreak the virus rapidly spread from one district to another or even in large cities, like Conakry (capital city of Guinea), Freetown (capital city of Sierra Leone), Monrovia (capital city of Liberia), and others. New cases arising in multiple locations make it difficult for response team to follow all signaled cases and even more difficult contact tracing activity. Since March 2014, when local and international authorities reported the start of an EVD outbreak in Guinea, all efforts were aimed at the containment of its spreading. This attempt to block the infectious disease at its beginning failed for many reasons that local and international partners have underestimated. Previous outbreaks did not cause problem of leadership, organization, or prevention: the isolation of the disease in rural areas and the low local community movement made it easy to manage. Contrariwise, in few months the current outbreak evolved in a large epidemic and one of the reasons may be a lack of leadership due to an underestimation of the disease.

The spread of EVD to several countries in such a short period is an unprecedented situation that needs to be faced with a unified strategy; cross border meetings and the Accra meeting in July 2014 have been fundamental to choose a common action plan to contain and stop the disease. The porous borders of affected countries forced authorities to increase collaboration with bordering countries to adopt common measures to effectively stop the infection. Under the leadership of WHO, an agreement has been reached on a common action plan which involves affected and bordering countries and the collaboration of international partners to reorganize resources and experts on the field in response to new cases detection on the affected countries. The response plan employed in each affected country demands a large number of resources and funds. The underestimation of the level of this epidemic caused a slow and insufficient financial and resources mobilization in local and international scale. Health personnel, experts, additional diagnostic capability, treatment facilities, vehicles to transport samples, and suspected cases are fundamental resources to improve this critical situation. The involvement of nations that can offer these resources is mandatory to avoid a further spread of the virus and limit the increasing number of the dead [48].

One of the first problems in the disease management has been the weak and unprepared health-care (HC) system. In fact none of HC workers had ever been involved in the management of cases of EVD, health security level was very low with the risk that HC personnel, not following security protocol, can become infected and spread the disease to other patients or colleagues; to avoid this problem HC workers training was one of the priorities in WHO and partners response strategy together with the deployment of laboratories for diagnosis and the creation of isolation facilities in the affected districts. The coordination between Minister of Health and international partners was and is essential for the response to EVD outbreak. To train and coordinate local health workers and response teams many international experts have been deployed to affected areas and personal protective equipment has been arranged to treatment facilities. Unfortunately the situation is worse than any prevision: the number of medical staff infected in West Africa till now is critical (240 including 120 deaths) [49], HC system is collapsing, in Liberia many treatment centers and general clinics have closed, the population have no access to basic HC, and fear of infection keeps patients out and causes medical staff to flee. Only a massive involvement of personnel and resources can improve the situation [50, 51].

Another relevant crisis has been the community reaction. The involvement of several countries in this EVD outbreak is due to many cross border movements between communities and mistaken belief about the origin of the disease. Alert on bordering countries was provided by WHO at early stage of this outbreak, but tracing of movements on a so large territory has proven to be a difficult challenge; first cases reported in Sierra Leone and Liberia were identified as people who travelled through an affected district of Guinea. Community collaboration to signal any diseased member or suspect death and to help health authorities to trace all possible contacts is fundamental to interrupt virus spreading; unfortunately there have been many cases in which community did not cooperate and health teams were forced to face the hostility of the population. In some areas, response teams discovered the existence of various “shadow zones,” location where medics are not allowed to enter, where cases and deaths cannot be calculated [19]. For example, as reported in an interview by MSF doctors on July 9, 2014, “There are still 20 villages around Guéckédou that continue to deny access to medical teams” [11]. Many people continue to think that health authorities are responsible for spreading the disease and decline anybody's help; their trust is in traditional culture and not in modern medicine. These episodes happened quite often and represent a serious problem for teams on the field. Another serious event happened recently when protesters broke into a clinic contaminating instruments and 17 patients escaped the quarantine [52]. Fortunately they have been traced and were returned back only 3 days later [53]. Community lack of trust in health authorities could bring to other similar episodes and represent a huge difficulty for disease containment. The disrespect of security protocols from health authorities is another example: one of the modes of spread of infection is through dead bodies which remain contagious for days after the death; it is common practice in these communities to wash accurately the bodies before burial, a close contact with an affected dead body can often result in Ebola virus infection, and the morgue can spread the disease to other people. These are the reasons why many efforts, made by the local and international partners, have been aimed to improve proper information on the disease, increase community awareness, spreading knowledge and inspiring trust among the population, because community collaboration is fundamental to control and contain Ebola virus spreading. In affected countries political delegations visited affected districts to build a relationship of trust with community leaders to increase their commitment on EVD response.

The fear of the emerging of new foci of EVD around the world is one of the main concerns. The diffusion of EVD in highly industrialized countries is actually very unlikely: a strong and well organized HC system, a large amount of resources and personnel, and the presence of emergency plans to promptly respond to a similar situation result in a very improbable spread of an Ebola-like infection. Furthermore the probability of transmission of EVD during air travel is very low because the transmission involves a direct contact with the infected person's bodily fluids. However as precaution all countries have been requested to strengthen the capability to detect and immediately contain new cases, while affected countries have to conduct exit screening of all individuals at international airports, seaports, and major land crossings, for symptoms consistent with EVD; positive matches should not be allowed to travel except for proper medical evacuation [39]. While WHO does not advice any ban for international travel or trade to affected countries, to prevent a further spread of EVD epidemic, unaffected countries of West Africa adopted different security measures:* Côte d'Ivoire* closed its land borders with EVD-affected West African neighbors Guinea and Liberia; Kenya Airways stopped flying to Sierra Leone and Liberia; Senegal closed its borders to travelers from Liberia, Guinea, and Sierra Leone; Cameroon closed all its land, sea, and air borders with Nigeria.

A new emerging problem caused by governments' measures for outbreak response is population riots: Liberian President Ellen Johnson Sirleaf imposed a nighttime curfew and quarantined two affected neighborhoods; people inside the quarantine areas cannot go to work; food supplying and basic HC are no longer guaranteed. The rage of the population caused by the current situation forced the employment of soldiers and police officers to suppress the clashes [54]. The situation can only get worse without an international intervention to support the population.

The critical spreading of the infection highlights the need of improvement of efforts to find an effective treatment or vaccine: EVD has a high case-fatality rate and there is no therapy or vaccine approved for its treatment [55]; infected symptomatic patients can receive supportive care only. To respond to this emergency an ethical committee valued the possible use of EDs which showed promising results on phase one experimentation. The meeting resulted in consensus for the use of some particular EDs under precise conditions. At the moment the administration of the ED ZMapp is in progress: three doctors infected with Ebola virus in Liberia seem to have a good response to the therapy with authorities' sources talking of “remarkable signs of improvement” [56]. Two of these doctors healed completely and were discharged from hospital, and the third one died after the treatment. However ZMapp is an ED and cannot be produced in a massive way on short term. International health authorities are reviewing all projects involved in the development of treatment or vaccines for Ebola virus that could be helpful for cure and control of the disease [57]. Only recently Japan offered to WHO supplies of a no FDA-approved antiviral drug called Favipiravir, which is designed for influenza virus but could be effective also for Ebola virus infections; the stock Japan can provide could be enough for 20000 patients [58].

The situation is far from being under control, the infection continues to spread, and health authorities do not seem to be able to stop the disease. International efforts, logistical and economical, are being improved demanding all nations participation: resources, experts, and funds are mandatory for EVD disease response plan; all local and international partners efforts employed till now are not enough to contain the virus spread that in the last update results in more than 7903 deaths [14].

In view of the experiences provided by the natural outbreaks such as the EVD one in West Africa, it is necessary to develop new systems and technologies to improve the detection of biological agents and new methodologies to manage the spread of pathogens [59–65].

## 5. Addendum

Due to the rapid evolution of the EVD outbreak, all the data shown in figures and/or table of this paper represents the situation up to August 15, 2014. Preliminary notices, included in reports between August 16 and August 31, were only cited into the text but not included in figures and/or tables.

## Figures and Tables

**Table 1:** Periods considered for time-to-time evaluation of the EVD outbreak during 2014 in West Africa. First column represents the period. The third column represents the data of the report released by WHO or CDC. In the fourth column the duration of each period is represented in days. The last column shows the days progression.

**1**	From	—	To	March 22	n.a.	0
**2**		March 23		April 1	9	10
**3**		April 2		April 13	11	22
**4**		April 14		April 24	10	33
**5**		April 25		May 5	10	44
**6**		May 6		May 14	8	53
**7**		May 15		May 27	12	66
**8**		May 28		June 9	12	79
**9**		June 10		June 21	11	91
**10**		June 22		June 30	8	100
**11**		July 1		July 14	13	114
**12**		July 15		July 23	8	123
**13**		July 24		July 30	6	130
**14**		July 31		August 7	7	139
**15**		August 8		August 15	7	145
